# Screening anti-fatigue components of American ginseng saponin by analyzing spectrum–effect relationship coupled with UPLC-Q-TOF-MS

**DOI:** 10.2478/abm-2023-0057

**Published:** 2023-10-18

**Authors:** Meiyu Lin, Shaiping Hu, Qi Zeng, Bixia Xiao, Yao He

**Affiliations:** School of Pharmacy, Hunan University of Chinese Medicine, Changsha, Hunan 410208, China; School of Pharmacy, Changsha Medical University, Changsha, Hunan 410209, China

**Keywords:** canonical correlation analysis, fatigue, mass spectrometry, *Panax quinquefolium* saponin, serum

## Abstract

**Background:**

American ginseng has an obvious anti-fatigue effect, but the effective material basis is still unclear. The spectrum–effect relationship is a scientific method that studies the correlations between chemical spectra and pharmacological effect.

**Objective:**

To reveal the real bioactive compounds in American ginseng saponin (AGS) based on a study of the underlying correlations between these compounds’ occurrence in rat serum after their intake of AGS and the anti-fatigue effect of AGS.

**Methods:**

We utilized ultra-performance liquid chromatography (UPLC) with quadrupole and time-of-flight mass spectrometry (Q-TOF-MS) to analyze the extract of AGS and its constituents in serum after oral administration in rats. The anti-fatigue effect of AGS in rats was measured using the time weight-bearing swimming technique, the content of blood urea nitrogen, hepatic glycogen, and blood lactic acid. The relationship between the peak area values in fingerprints from rat serum and pharmacodynamic parameters of AGS was established using correlation analysis with partial least square regression (PLSR) method and gray correlation method.

**Results:**

We detected and identified 22 compounds from extract, and 8 prototype components from serum. Through PLSR and gray correlation method, it was found that the ginsenosides Re, Rb1, and Rb2 were significantly positively related to the pharmacodynamic data.

**Conclusions:**

Based on the spectrum–effect relationship, PLSR and gray correlation method can be used to screen for the anti-fatigue components available in AGS. Such an approach is of practical significance as it provides an effective means for exploring the material basis for the efficacy of American ginseng, particularly as an anti-fatigue agent.

American ginseng is the dried root of *Panax quinquefolium L* [1]. It is said that it possesses medicinal properties that could exert a therapeutic influence on various biological activities, such as effects on the cardiovascular and central nervous systems, anti-tumor, immunomodulation, anti-fatigue, and anti-oxidation effects, etc. [2]. American ginseng is favored by people as a dietary health supplement in daily life. As we know, saponins are seen as the essential active components. At present, scientists separate and identify hundreds of ginsenosides from American ginseng, and a fair number of those have pharmacological properties [3]. American ginseng has lots of pharmacological actions but the effective material basis is still unclear. The efficacy of traditional Chinese medicine arises from not only the simple addition of each component but also the synergistic effect of various active substances [4]. In addition, the characteristics of multi-composition and multi-target of traditional Chinese medicine increase the difficulty of studying the basis of medicinal substances [5]. Therefore, a combined and scientific approach is needed to elucidate the relationship between ingredients and pharmacodynamics. The spectrum–effect relationship is a valid way to clarify the pharmacodynamic base for the effect [6]. Bioactive compounds must be absorbed into blood and then transferred to the target. So, only those components that are absorbed into the bloodstream are effective after oral administration [7]. Our study was conducted with the objective of ascertaining the chemical composition of compounds prevalent in rat serum after the administration of American ginseng saponin (AGS) that had the highest correlation with AGS's anti-fatigue effect, and thus to forge the underlying links between the fingerprint in serum and the anti-fatigue effect of AGS. Our results could provide a reference for the research of the pharmacodynamic substantial foundation of AGS.

## Methods

Standards of ginsenosides Re (catalog No. 110754-201525), Rb1 (catalog No. 110704-201424), Rb2 (catalog No. 111715-201203), Rd (catalog No. 111818-201302), and pseudoginsenoside-F11 (catalog No. 110841-201406) were bought from China National Institutes for Food and Drug Control. Samples of ginsenosides Rc, Rb3, and Rg1 were provided by College of Chemistry, Jilin University. The purity of all the standards was above 98.0%. Methanol and acetonitrile (Thermo Fisher Scientific) and formic acid (CNW Technologies GmbH) were high performance liquid chromatography-tandem mass spectrometry (HPLC-MS) grade. Other chemicals and reagents were analytical grade or above. The American ginseng used in the present study (Beijing Tongrentang Pharmacy) has its origin in Wisconsin, USA. It was identified as *Panax quinquefolium L* by Professor Zhou Xiaojiang of Hunan University of Chinese Medicine. We used assay kits for determination of hepatic glycogen (HG), blood urea nitrogen (BUN), and blood lactic acid (LA) (Nanjing Jiancheng Biotechnology Institute).

Thirty SPF Sprague Dawley rats (Hunan SJA Laboratory Animal Company), weighing 200–220 g, were housed in groups of 5 animals per cage under a constant temperature (23 ± 2 ºC) and humidity (50% ± 10%) on a 12 h light–dark cycle. The present animal experiment is in conformity with the Regulations of Experimental Animal Administration, and was approved by the State Committee of Science and Technology of the People's Republic of China (certificate of approval No. 201805220003).

### Preparation of AGS and standard solution

**Figure 1.**
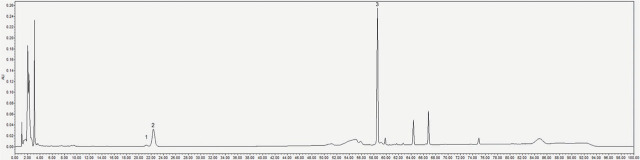
The HPLC chromatogram of AGS. The total content of ginsenosides Rg1, Re, and Rb1 was measured as 2.3% using HPLC. According to the provisions of Chinese Pharmacopoeia 2015, the total amount of ginsenosides Rg1, Re, and Rb1 should not be less than 2.0%. The content was up to the standards, indicating that the samples were qualified: 1, ginsenoside Rg1; 2, ginsenoside Re; and 3, ginsenoside Rb1. AGS, American ginseng saponin.

The AGS was processed according to the Chinese Pharmacopoeia 2015 [8]. The decoction pieces of American ginseng were boiled for 2 times, 2 h for the first time, and for 1.5 h for the second time. Water 10 times the weight of American ginseng was added each time. The filtrates of the two times were combined. The filtrate was eluted with water till it became colorless through D101 macroporous adsorption resin column, and then eluted with 60% ethanol. The 60% ethanol was concentrated into the extract, after which it was dried and crushed. The content of AGS was measured using vanillin–glacial acetic acid–perchloric acid in a UV-2550 ultraviolet spectrophotometer (Shimadzu) [9]. The total saponin content of American ginseng was 81%. The total content of ginsenosides Rg1, Re, and Rb1 was determined using HPLC (e2695, Waters) [8]. Acetonitrile was used as mobile phase A, and 0.1% phosphoric acid solution as mobile phase B. The gradient elution condition was performed, as follows: 0–25 min, 19%–20% A; 25–60 min, 20%–40% A; 60–90 min, 40%–55% A; 90–100 min, 55%–60% A. The detection wavelength was 203 nm. The column temperature was 40 ºC. The total content of ginsenosides Rg1, Re, and Rb1 was 2.3%, which was up to the Chinese Pharmacopoeia 2015 standards. The HPLC chromatogram is shown in **Figure 1**. Accurately weighed AGS sample powders (20 mg) were dissolved in 25 mL methanol.

A certain amount of standards was dissolved in methanol in numerous instances to produce various standard stock solutions. Adding different amounts of the above-mentioned standard stock solutions makes the mixed standard solution obtain final concentrations of about 1.50 μg/mL for each standard substance.

All the solutions were kept in reserve at 4 ºC in the pre-analytical phase. Before UPLC-Q-TOF-MS analysis, all the solutions were filtered through a 0.22 μm microporous membrane.

### UPLC-Q-TOF-MS analysis

Chromatographic analysis was conducted with the application of a Waters ACQUITY UPLC I-Class system, provided with an autosampler, a thermostated column compartment, and a binary solvent delivery system. ACQUITY BEH C18 (2.1 mm × 100 mm, 1.7 μm, Waters) was for chromatographic fractionation. The mobile phase is composed of 0.1% formic acid in Milli-Q water (A) and acetonitrile (B). The current velocity of it stood at 0.3 mL/min. The UPLC elution condition is: 0–3 min, 20%–21% B; 3–5 min, 20%–21% B; 5–7 min, 22%–23% B; 7–10 min, 23%–25% B; 10–11 min, 25%–28% B; 11–17 min, 28%–30% B;17–22 min, 30%–35% B; 22–27 min, 35%–60% B; 27–31 min, 60%–90% B; 31–32 min, 90%–100% B; 32–36 min, 100% B; 36–42 min, 100%–20% B. The column temperature was 35 ºC, and the injection volume was 5 μL.

A Waters Xevo G2-XS Q-TOF system was offered for the MS detection. An electrospray ionization source (Waters) was available. All the operation and data analyses were conducted using Masslynx V4.1 software (Waters). The mass spectrometry was performed in the negative mode, and the optimized mass parameters were as below: capillary voltage, 2.5 kV; extraction cone voltage, 4 V; sample cone voltage, 40 V; source temperature, 120 ºC; cone gas flow rates, 50 L/h; desolvation gas flow rates, 500 L/h; and desolvation temperature, 600 ºC. Data were gathered in continuum mode. The width is 100–1200 Da. A lock-mass of leucine enkephalin was used in mass spectrometry analysis to ensure accuracy.

### Data acquisition and processing

Liquid chromatography (LC) and mass spectrometry (MS) data were preprocessed with MarkerLynx V4.1 software for peak detection and peak alignment. Researchers retrieved the data by performing detailed searches in databases. The National Medical Library PubMed and the National Institutes of Health, SciFinder scholars of the American Chemical Society, and China National Knowledge Infrastructure (CNKI) of Tsinghua University were reported in the literature from a review of *Panax* species in Microsoft Office Excel. The sheet established a data bank that contains the name, molecular formula, chemical structure, and literature for each chemistry. The above information was imported into the UNIFI Scientific Information System (Waters) in the form of a database. UNIFI can identify compounds through an automated matching database. The “Search Database” in the “Identify Compounds” in UNIFI was used for qualitative analysis of chromatographic peaks.

### Animal assays

Rats were randomized into two groups; blank group and fatigue model group. Model group rats were further randomized to negative model group and test group. The rats in test group were orally administrated with AGS at a potion of 50 mg/kg once per day and successively for 30 d; meanwhile, the rats in blank and negative model group were given water instead. There were 10 rats in each group. The rats in the fatigue model group were exposed to weight-loaded swimming every day. On the final day, all the rats were forced to swim after administration. It was determined that the rats were exhausted when they lacked normal coordination, and failed to surface in 10 s [10]. Researchers kept track of exhaustive swimming time at once. The next day, blood was collected in the second hour after administration. Serum samples were prepared for the BUN and LA determination and the livers for HG determination. All biochemical parameters were measured using commercial kits, and the whole of the process was performed in accordance with the instruction manual. All empirical data were represented as mean ± standard deviation and *T* test was applied. The data were analyzed for frequency and tested for significance using the statistical software SPSS20.0 (International Business Machines Corporation, IBM) computer package.

### Serum sample preparation for serum pharmacochemistry

Blood specimens were taken from the retinal venous plexus from the rats in the test group before the administration of AGS. The blood samples, together with those collected after the animal experiments, were kept for 30 min and then centrifuged at 4,000 rpm for 10 min. Next, 400 μL methanol was added to the 100 μL serum specimens, followed by thorough mixing for 5 min, and being centrifuged at 12,000 rpm at 4 ºC for 10 min. The supernatant was evaporated to dryness with a frozen dryer, re-dissolved in 100 μL methanol, and centrifuged at 12,000 rpm for 10 min at 4 ºC; thereafter, the supernatant was injected into the LC-MS system for research purpose.

### Spectrum–effect relationship

**Figure 2.**
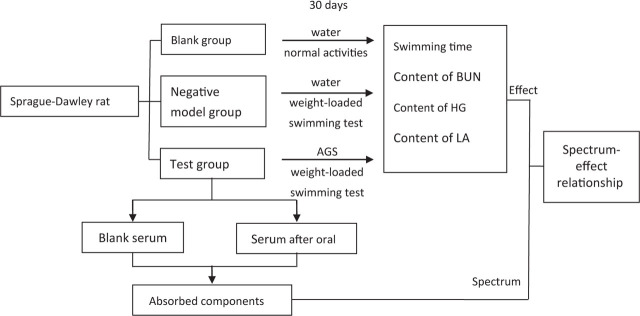
The flow chart of the study. Rats were randomized into three groups: blank group, negative model group, and test group. The rats in the test group were orally administrated with AGS at a portion of 50 mg/kg once per day and successively for 30 d; meanwhile, the rats in the blank and negative model groups were given water instead. The anti-fatigue effect of AGS was measured based on the swimming time, as well as on the contents of BUN, HG, and LA in rats. Blood specimens were taken from the retinal venous plexus from the rats in the test group both before and after administration of AGS. To identify the components of AGS that had undergone absorption in serum, measurements were made after administration, and the measured values were compared with the corresponding values obtained before administration. The relationship between the peak area values from rat serum and pharmacodynamic parameters of AGS was established using PLSR and gray correlation method. AGS, American ginseng saponin; BUN, blood urea nitrogen; HG, hepatic glycogen; LA, lactic acid.

The partial least squares regressions (PLSR) and gray correlation method were carried out using MATLAB 2017b (MathWorks Inc.) to analyze the correlation between the values of peak areas in LC-MS fingerprints and pharmacological results from the anti-fatigue experiment. The flow chart of this study is as shown in **Figure 2**.

## Results

### Chemical analysis of AGS

**Figure 3.**
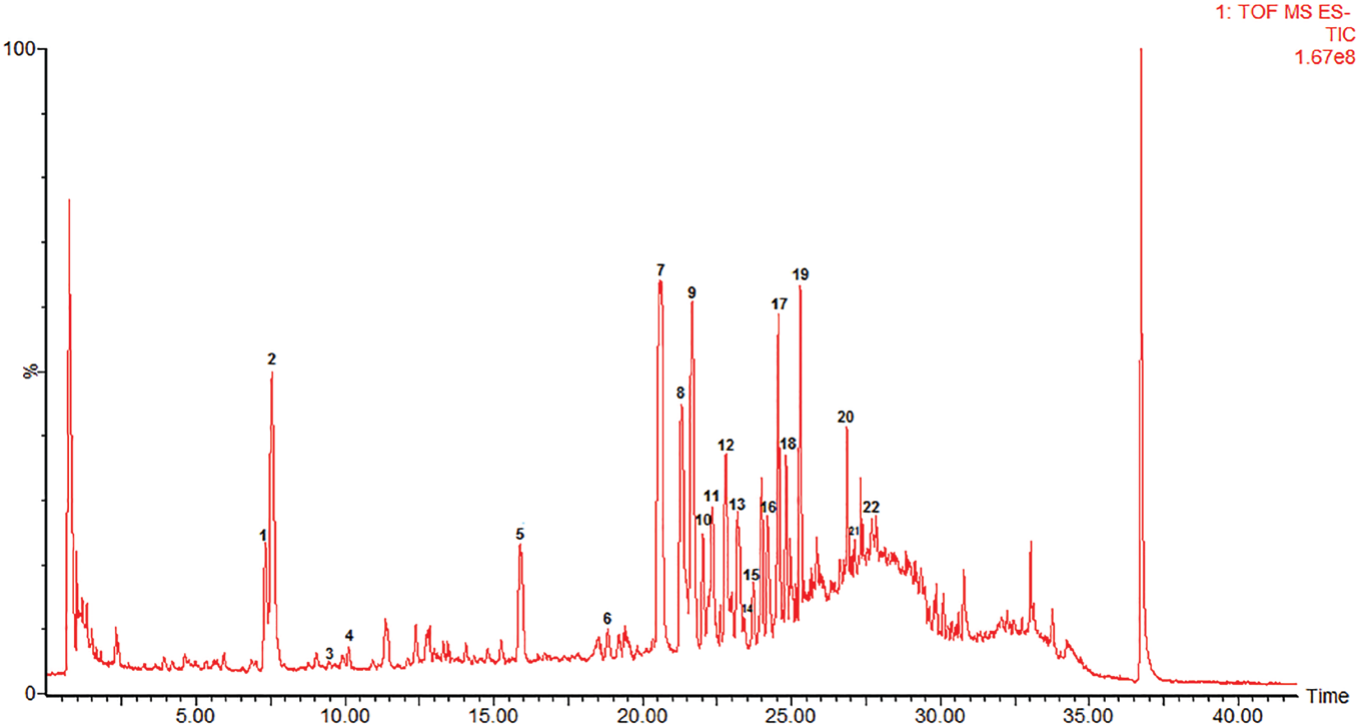
TIC of AGS. UPLC-Q-TOF-MS in negative ESI mode was utilized to analyze the extract of AGS: 1, ginsenoside Rg1; 2, ginsenoside Re; 3, ginsenoside Malonyl-Rg1; 4, ginsenoside Malonyl-Re; 5, pseudoginsenoside-F11; 6, 20(S)-ginsenoside Rg2; 7, ginsenoside Rb1; 8, malonylginsenoside Rb1; 9, ginsenoside Rc; 10, malonylginsenoside Rb1 isomer; 11, malonylginsenoside Ra2; 12, ginsenoside Rb2; 13, ginsenoside Rb3; 14, malonylginsenoside Rb2 isomer; 15, malonylginsenoside Rb3 isomer; 16, Zingibroside R1; 17, ginsenoside Rd; 18, malonylginsenoside Rd; 19, gypenosid XVII; 20, 20(S)-ginsenoside Rg3; 21, Chikusetsu saponin IVa; 22, 20(R)-ginsenoside Rg3. AGS, American ginseng saponin.

The results obtained from carrying out a Total ion chromatography (TIC) of AGS using UPLC-Q-TOF-MS in negative electrospray ionization (ESI) mode are displayed in **Figure 3**. The compositions of AGS were well-separated and identified by analyzing the precise relative molecular mass of primary mass spectrometry, the cleavage fragments of MS/MS and reported paper [11,12,13]. Twenty-two peaks were identified, and the results are shown in **Table 1**.

**Table 1.** Identification of composition of AGS using UPLC-Q-TOF-MS

**No.**	**t_R_/min**	**Molecular formula**	**Mean measured mass**	**Theoretical exact mass**	**δ**	**[M − H + HCOOH]^−^**	**Identity**	**Fragments**
1	7.33	C_42_H_72_O_14_	799.4908	799.4849	7.38	845.5096	Ginsenoside Rg1	637.4366 [M–Glc–H]^−^ 475.3792 [M–2Glc–H]^−^
2	7.55	C_48_H_82_O_18_	945.5429	945.5428	0.11	991.5691	Ginsenoside Re	799.4850 [M–Rha–H]^−^ 783.4913 [M–Glc–H]^−^ 637.4314 [M–Rha–Glc–H]^−^
3	9.48	C_45_H_74_O_17_	885.4820	885.4853	−3.72	–	Ginsenoside Malonyl-Rg1	841.4949 [M–CO_2_–H]^−^
4	10.14	C_51_H_84_O_21_	1031.5400	1031.5432	−3.10	–	Ginsenoside Malonyl-Re	987.5555 [M–CO_2_–H]^−^
5	15.87	C_42_H_72_O_14_	799.4850	799.4844	0.75	845.4918	Pseudoginsenoside-F11	653.4298 [M–Rha–H]^−^
6	18.80	C_42_H_72_O_13_	783.4913	783.4900	1.66	829.4960	20 (S)-ginsenoside Rg2	637.4314 [M–Xyl–H]^−^ 475.3792 [M–Xyl–Glc–H]^−^
7	20.58	C_54_H_92_O_23_	1107.5995	1107.5957	3.43	1153.6097	Ginsenoside Rb1	945.5552 [M–Glc–H] − 783.4336 [M–2Glc–H]^−^
8	21.32	C_57_H_94_O_26_	1193.6053	1193.5961	7.71	–	Malonylginsenoside Rb1	1149.6067 [M–CO_2_–H]^−^
9	21.67	C_53_H_90_O_22_	1077.5851	1077.5845	0.56	1123.5918	Ginsenoside Rc	945.5429 [M–Araf–H]^−^ 783.4913 [M–Araf–Glc–H]^−^
10	22.02	C_57_H_94_O_26_	1193.5983	1193.5961	1.84	–	Malonylginsenoside Rb1 isomer	1149.6136 [M–CO_2_–H]^−^
11	22.34	C_56_H_92_O_25_	1163.5919	1163.5855	5.50	–	Malonylginsenoside Ra2	1119.5933 [M–CO_2_–H]^−^
12	22.79	C_53_H_90_O_22_	1077.5851	1077.5845	0.56	1123.5918	Ginsenoside Rb2	945.5429 [M–Arap–H]^−^ 915.5326 [M–Glc–H]^−^ 783.4913 [M–2Glc–H]^−^
13	23.18	C_53_H_90_O_22_	1077.5851	1077.5845	0.56	1123.5918	Ginsenoside Rb3	945.5429 [M–Xyl–H]^−^ 915.5326 [M–Glc–H]^−^ 783.49133 [M–2Glc–H]^−^
14	23.41	C_56_H_92_O_25_	1163.5850	1163.5849	0.086	–	Malonylginsenoside Rb2 isomer	1119.6001 [M–CO_2_–H]^−^
15	23.72	C_56_H_92_O_25_	1163.5919	1163.5849	6.02	–	Malonylginsenoside Rb3 isomer	1119.6069 [M–CO_2_–H]^−^
16	24.20	C_42_H_65_O_14_	793.4396	793.4374	2.8	–	Zingibroside R1	631.38606 [M–Glc–H]^−^
17	24.56	C_48_H_82_O_18_	945.5429	945.5428	0.11	991.5626	Ginsenoside Rd	783.4913 [M–Glc–H]^−^ 621.4373 [M–2Glc–H]^−^
18	24.81	C_51_H_84_O_21_	1031.5466	1031.5432	0.58	–	Malonylginsenoside Rd	987.5684 [M–CO_2_–H]^−^
19	25.29	C_48_H_82_O_18_	945.5429	945.5423	0.63	991.5626	Gypenosid XVII	783.4913 [M–Glc–H]^−^ 621.4373 [M–2Glc–H]^−^
20	26.87	C_42_H_72_O_13_	783.4913	783.4900	1.66	829.4960	20 (S)-ginsenoside Rg3	621.4375 [M–Glc–H]^−^ 459.4088 [M–2Glc–H]^−^
21	27.12	C_42_H_66_O_14_	793.4396	793.4380	2.02	–	Chikusetsu saponin IVa	613.3718 [M–Glc–H]^−^
22	27.69	C_42_H_72_O_13_	783.4913	783.4900	1.66	829.5065	20 (R)-ginsenoside Rg3	621.4373 [M–Glc–H]^−^

AGS, American ginseng saponin.

### Identification of components in serum samples

**Figure 4.**
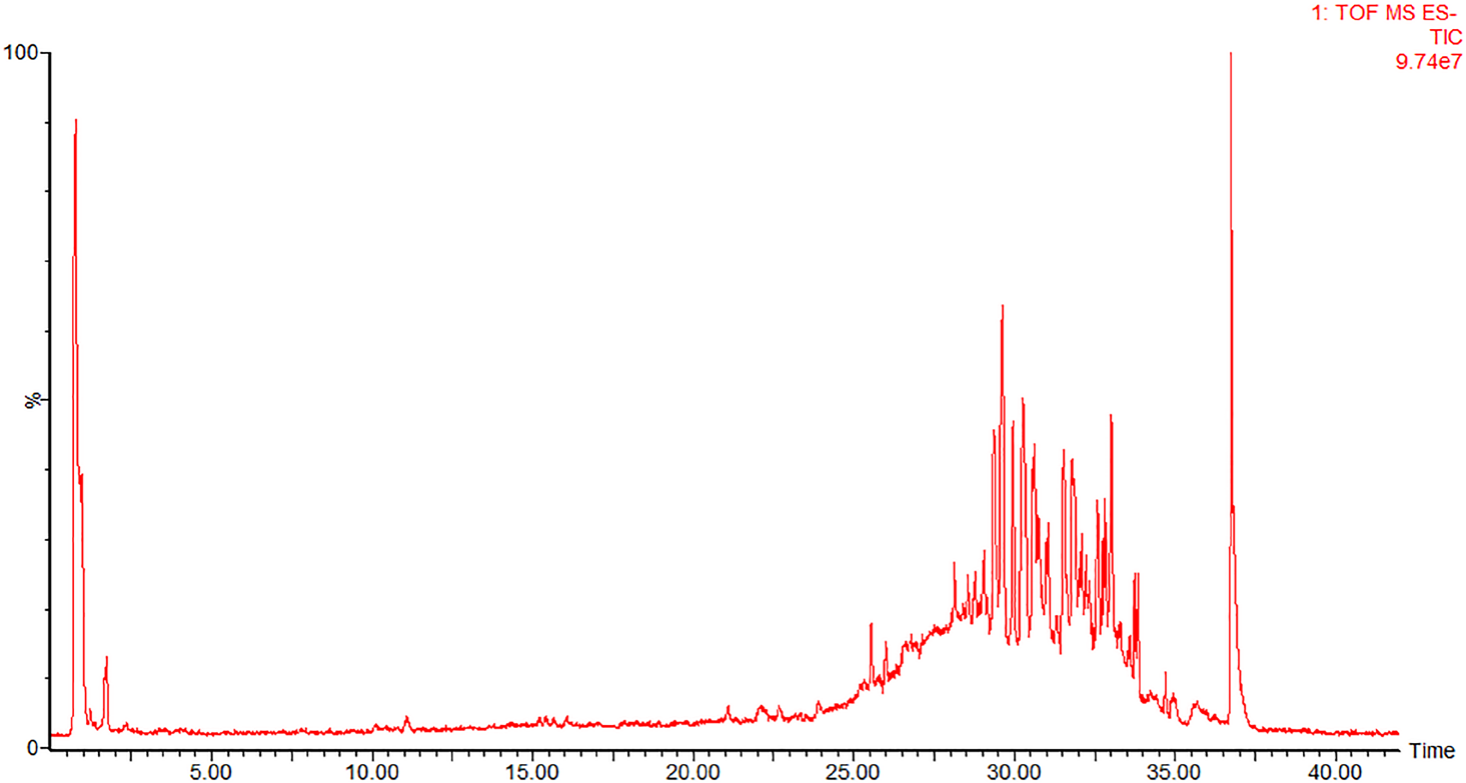
TIC of blank serum. UPLC-Q-TOF-MS in negative ESI mode was utilized to analyze blank serum from rat before administration of AGS. AGS, American ginseng saponin.

**Figure 5.**
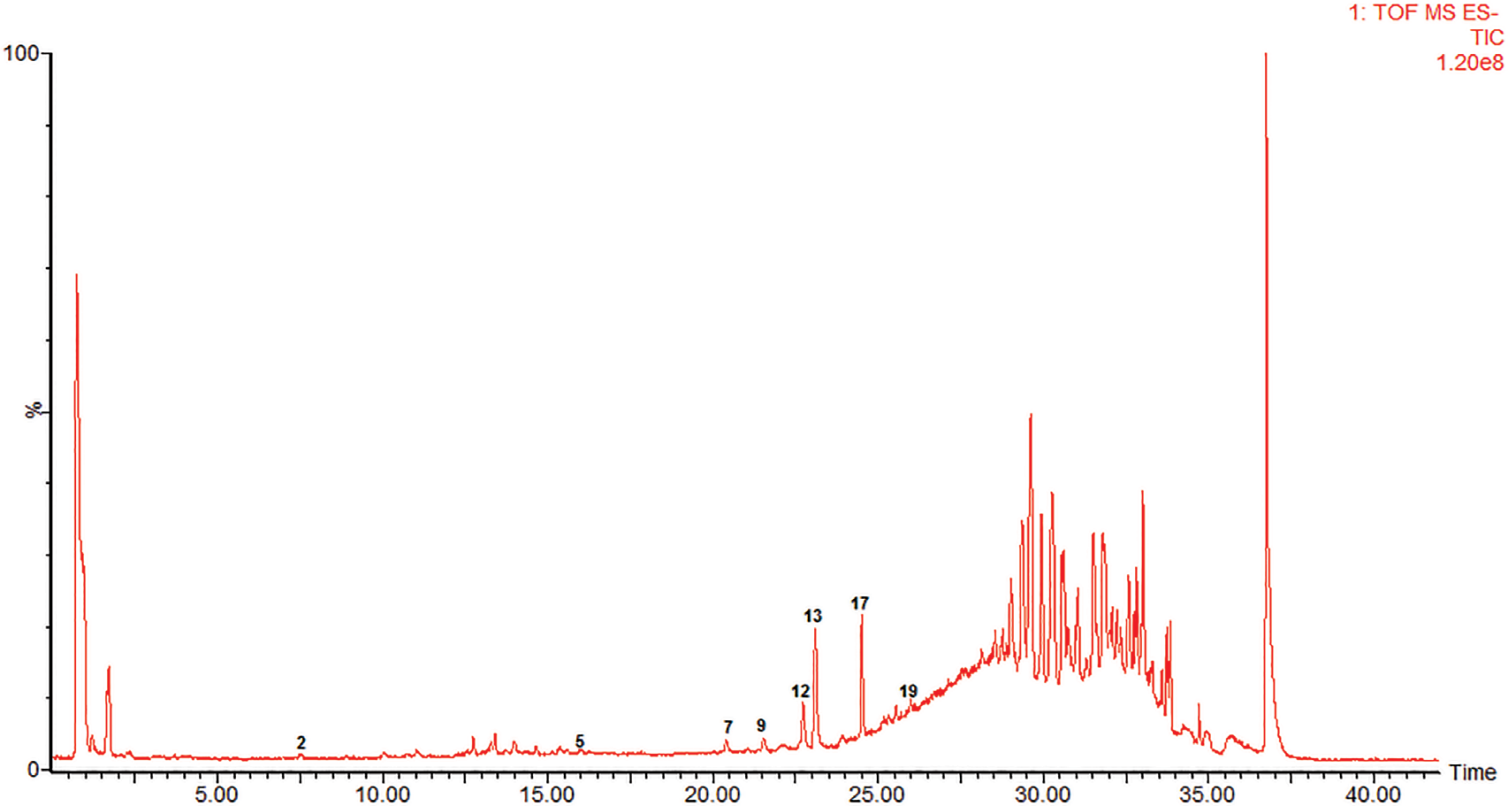
TIC of serum containing drug. UPLC-Q-TOF-MS in negative ESI mode was utilized to analyze serum containing drug from rat after administration of AGS: 2, ginsenoside Re; 5, pseudoginsenoside-F11; 7, ginsenoside Rb1; 9, ginsenoside Rc; 12, ginsenoside Rb2; 13, ginsenoside Rb3; 17, ginsenoside Rd; and 19, gypenosid XVII. AGS, American ginseng saponin.

Eight components were detected in the serum samples after administration, compared with the serum collected before administration. The TIC of blank serum and serum containing drug, obtained with the use of UPLC-Q-TOF-MS in negative ESI mode, are, respectively, displayed in **Figures 4 and 5**. Through comparisons with the standard as well as with the literature, they were identified as ginsenoside Re, pseudoginsenoside-F11, ginsenosides Rb1, Rc, Rb2, Rb3, and Rd, and gypenosid XVII. They are all prototype components.

### Anti-fatigue activity

The rats were weighed daily and were given appropriate medication. No adverse events occurred in any of the groups. As shown in **Table 2**, swimming time and content of HG in the test group outclassed those in the negative model group; meanwhile, the contents of BUN and LA were well below those in the negative model group.

**Table 2.** Anti-fatigue effect of AGS

**Group (n = 10)**	**Swimming time (min)**	**Content of BUN (mmol/L)**	**Content of HG (mg/g)**	**Content of LA (mmol/L)**
Blank group	57.8 ± 8.6	8.57 ± 1.03	7.21 ± 1.05	7.62 ± 0.75
Negative model group	65.7 ± 9.5	8.28 ± 1.25	7.31 ± 1.24	7.39 ± 0.90
Test group	122.3 ± 18.9^***^	7.21 ± 1.14^*^	12.81 ± 1.58^***^	6.42 ± 0.75^*^

* *P* < 0.05,

** *P* < 0.01,

*** *P* < 0.001 vs negative model group.

AGS, American ginseng saponin; BUN, blood urea nitrogen; HG, hepatic glycogen; LA, lactic acid.

### Analysis of spectrum–effect relationship

The eight common peak areas were marked as independent variables (X). Swimming time, contents of HG, BUN, and LA were marked as dependent variables (Y). Regression and correlation analyses were constituted using PLSA analysis. Employing these values, we derived correlation coefficients. The results of the data were as follows:

Swimming time
  Y1 = 0.02 × 2–0.03 × 5 + 0.05 × 7–0.05 × 9 + 0.03 × 12 + 0.18 × 13 + 0.19 × 17-0.03 × 19
Content of BUN
  Y2 = 0.01 × 2 + 0.01 × 5 + 0.02 × 7 + 0.02 × 9 + 0.01 × 12–0.02 × 13-0.01 × 17 + 0.01 × 19
Content of HG
  Y3 = 0.05 × 2–0.04 × 5 + 0.08 × 7–0.09 × 9 + 0.02 × 12 + 0.18 × 13 + 0.23 × 17-0.06 × 19
Content of LA
  Y4 = 0.05 × 2 + 0.08 × 5 + 0.03 × 7 + 0.02 × 9 + 0.11 × 12 + 0.15 × 13 + 0.25 × 17 + 0.05 × 19

**Figure 6.**
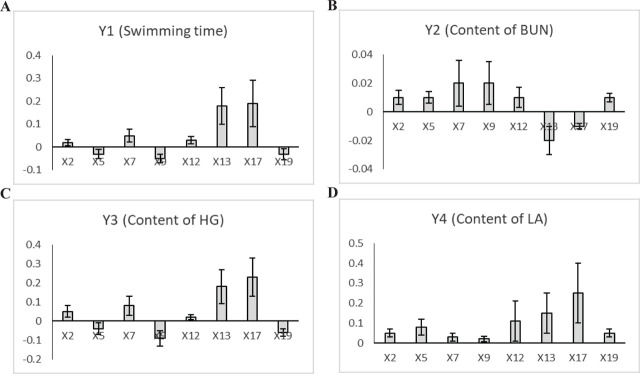
The regression coefficients plot of pharmacological results. The coefficient value before X reflects the degree of contribution of X to Y. **(A)** Using the regression equation coefficients, the graphs of regression coefficients of swimming time have been drawn. **(B)** Using the regression equation coefficients, the graphs of regression coefficients of the content of BUN have been drawn. **(C)** Using the regression equation coefficients, the graphs of regression coefficients of the content of HG have been drawn. **(D)** Using the regression equation coefficients, the graphs of regression coefficients of the content of LA have been drawn. Values are shown as mean (n = 10); error bars indicate standard deviation. BUN, blood urea nitrogen; HG, hepatic glycogen; LA, lactic acid.

The coefficient value before X reflects the degree of contribution of X to Y. Using the regression equation coefficients, the graphs of regression coefficients of physiological activity have been drawn (**Figure 6**).

Gray correlation theory modeling was used to analyze the correlation degree of eight common peak areas with swimming time and contents of HG, BUN, and LA. The results are shown in **Table 3**.

**Table 3.** Correlation between 8 common peak areas and efficacy

**No.**	**Swimming time**	**Content of BUN**	**Content of HG**	**Content of LA**
2	0.91 ± 0.04†	0.90 ± 0.06	0.94 ± 0.05	0.95 ± 0.04
5	0.70 ± 0.22	0.80 ± 0.13	0.70 ± 0.21	0.70 ± 0.21
7	0.95 ± 0.05	0.93 ± 0.05	0.95 ± 0.06	0.93 ± 0.06
9	0.64 ± 0.24	0.72 ± 0.17	0.63 ± 0.22	0.63 ± 0.22
12	0.86 ± 0.10	0.85 ± 0.11	0.89 ± 0.11	0.88 ± 0.12
13	0.86 ± 0.10	0.69 ± 0.22	0.89 ± 0.10	0.87 ± 0.11
17	0.83 ± 0.10	0.62 ± 0.22	0.85 ± 0.13	0.84 ± 0.13
19	0.75 ± 0.19	0.83 ± 0.10	0.74 ± 0.17	0.74 ± 0.17

† X = mean ± standard deviation.

BUN, blood urea nitrogen; HG, hepatic glycogen; LA, lactic acid.

## Discussion

UPLC-Q-TOF-MS is applied for fast analysis and identification of chemical constituents. It is characterized by high efficiency, wide quality range, high ion transport efficiency, high resolution, high sensitivity, and good reproducibility. In ESI negative mode, the characteristic fragments are obvious, which is beneficial to the structure analysis.

Both serum pharmacochemistry and anti-fatigue experiments were conducted for the test group, which can reduce the use of animals. In addition, it is more convincing to study the spectrum–effect relationship by detecting the absorbed components in serum and efficacy of the same animal.

The bioactive component of the drug in vivo is the drug transitional component in the serum after the drug administration. The analysis and search for the components that were prevalent in rat serum after the oral administration can provide a means for the elucidation of the basis of its pharmacodynamic substance. Therefore, not only the components of the AGS extract but also the components absorbed from AGS into rat serum were analyzed. In the process of serum pharmacochemistry experiments, the time of blood collection was explored, with the goal of obtaining more peak information, and the time of blood collection was optimized for 2 h of drug administration. In addition, the pretreatment method of serum samples was optimized, and the solid-phase extraction, liquid–liquid extraction, and protein precipitation were tried. Among them, use of the methanol precipitation method resulted in obtaining of the most abundant serum characteristic information, and the operation was simple and fast. Eight prototypes constituents were detected and identified.

As shown in the results of anti-fatigue activity, there was obvious discrepancy between the test group and the negative model group. After vigorous exercise, the liver glycogen will be used up, and the energy comes from the circulating glucose set free by the liver. So, adding the HG reserves is helpful in improving stamina and athletic ability [14]. When the body lacks energy, it will consume protein and BUN will rise. The decrease of BUN indicates that the body is more adaptable to fatigue and can speed up banishing fatigue [15]. LA is the glycolysis outcome of carbohydrate and will damage some organs and cause fatigue. AGS can restrain the LA accumulation in muscles and accelerate the elimination of LA, thus delaying fatigue [16]. Based on the above, AGS has obvious anti-fatigue effect.

In the light of the PLSA outcome, peaks 2, 7, 12, 13, and 17 revealed a positive correlation with the swimming time. Peaks 2, 5, 7, 9, 12, and 19 revealed a positive correlation with the content of BUN. Peaks 2, 7, 12, 13, and 17 revealed a positive correlation with the content of HG. Peaks 2, 5, 7, 9, 12, 13, 17, and 19 revealed a positive correlation with the content of LA. The intersection of regression and the results of correlation analysis were the peaks 2 (ginsenoside Re), 7 (ginsenoside Rb1), and 12 (ginsenoside Rb2). Previous studies have shown that ginsenosides Re, Rb1, and Rb2 can delay fatigue generation and promote fatigue recovery [17,18,19], which is consistent with the results of the present research. The interaction among them and the mechanism for it are still uncertain.

According to the results of gray correlation method, the compounds associated with swimming time were in the order of peaks 7, 2, 12, 13, 17, 19, 5, and 9. The compounds related to content of BUN were in the order of peaks 7, 2, 12, 19, 5, 9, 13, and 17. The order of compounds related to content of HG was peaks 7, 2, 12, 13, 17, 19, 5, and 9. The order of correlation with content of LA was peaks 2, 7, 12, 13, 17, 19, 5, and 9. The correlations between peaks 2 (ginsenoside Re), 7 (ginsenoside Rb1), and 12 (ginsenoside Rb2) and the four pharmacodynamic indicators were all greater than 0.85. This result was consistent with the PLSR outcome mentioned above.

The research on spectrum–effect relationship mainly includes principal component analysis, multiple linear regression, cluster analysis, gray correlation method, and neural network [20]. In the present study, the spectrum–effect relationship was studied using PLSA and gray correlation method. The data model of PLSA will include all the primitive independent variables, which can not only make the best use of the data information but also realize regression modeling (multiple linear regression), data structure simplification (principal component analysis), and related analysis between the two groups of variables (typical correlation analysis). Gray correlation is a method to describe the degree of correlation among factors through the order of gray correlation degree, which can make full use of the original information of dynamic indicators. The multivariate correlation analysis solved using gray correlation degree method is suitable for solving the correlation analysis of multi-fingerprint peak and pharmacodynamic activity directly in the spectrum–effect relationship. Both PLSA and gray correlation method can effectively analyze pharmacodynamic substances, and their results provide direct confirmation of each other's.

## Conclusion

An efficient UPLC-Q-TOF-MS was utilized to analyze AGS extract, and to assess the AGS components absorbed into the rat serum after the administration of AGS. Totally, 22 compounds from extract and 8 prototypes constituents from serum were detected and identified. The spectrum–effect relationship between AGS constituents in rat serum and the anti-fatigue effect of AGS was established using PLSA and gray correlation method. The experiment's result indicated that the anti-fatigue physiological activity of AGS was a comprehensive representation of the mixture of compounds, and that the anti-fatigue components of AGS are ginsenosides Re, Rb1, and Rb2. In short, this project offers a valid way for the screening of active constituents and reference for further study.
